# Pseudoaneurysm and Renal Artery Stenosis Post-renal Transplant: A Rare Presentation

**DOI:** 10.7759/cureus.47315

**Published:** 2023-10-19

**Authors:** Nashwan A Alattab, Yasir Suliman, Tariq M Wani, Khalid A Alhazmi, Abdulhakim I Bin Onayq, Saleh T Mahjoub

**Affiliations:** 1 Vascular Surgery, King Fahad Medical City, Riyadh, SAU; 2 Vascular Surgery, Burjeel Medical City, Abu Dhabi, ARE; 3 Surgery, Umm Al-Qura University, Makkah, SAU; 4 Medicine and Surgery, King Saud University Medical City, Riyadh, SAU

**Keywords:** case report, pseudoaneurysm, renal artery stenosis, graft dysfunction, hypertension, renal transplantation

## Abstract

We report the case of a 51-year-old gentleman who underwent living renal transplantation in Pakistan for end-stage renal disease one and a half years ago. He presented to our hospital with renal artery stenosis and an extra-renal pseudoaneurysm at the anastomotic site of the transplanted kidney. This can cause graft dysfunction and hypertension due to impairment of arterial perfusion in the transplanted kidney. Treatment with percutaneous transluminal angioplasty and covered stenting of the pseudoaneurysm and stenosis improved kidney function and hypertension.

## Introduction

Vascular complications post-renal transplantation are commonly reported, ranging from as low as 3% to 30% [1]. Based on the variable definitions of vascular complications and the imaging techniques used for their diagnosis, there is a wide range in the literature [2]. Transplant renal artery stenosis (TRAS), renal vein or artery thrombosis, hematoma, and pseudoaneurysm formation are common vascular complications post-renal transplantation [3]. TRAS is by a great margin the most common vascular complication after renal transplantation. TRAS has been identified as a cause of post-transplant hypertension, graft dysfunction, and graft loss [2]. The introduction of a color duplex has made the diagnosis of TRAS easier, with its incidence increasing to 12% [4]. Pseudoaneurysm post-renal transplant (PAPRT) is a rare complication with an incidence rate of less than 0.5% [5-7]. PAPRT is potentially life-threatening because sudden rupture can lead to torrential hemorrhage; thus, it needs to be managed aggressively by surgical excision or even graft nephrectomy [8]. Here, we report a case of post-renal transplantation presenting with TRAS concomitantly with a pseudoaneurysm.

## Case presentation

**Figure 1:**
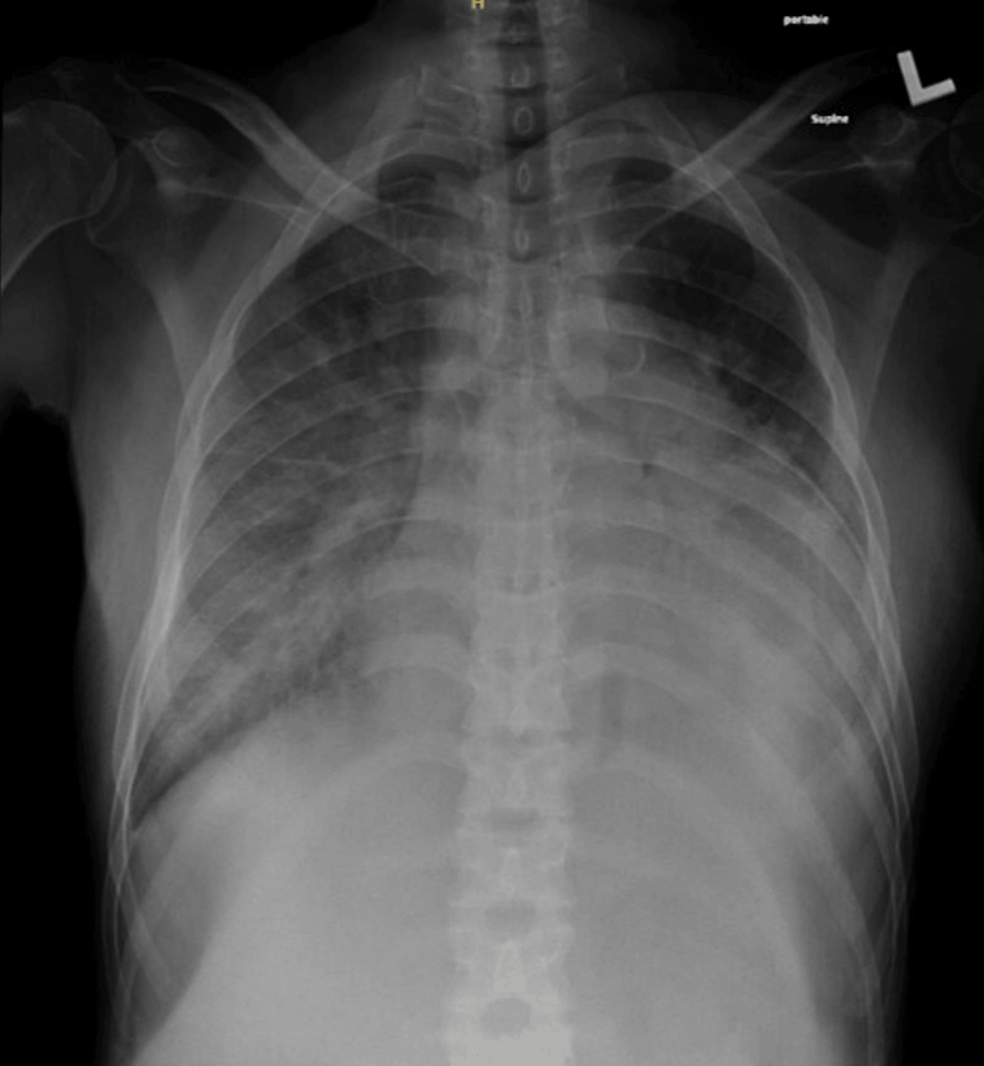
Anteroposterior chest X-ray showing flush pulmonary edema.

We report the case of a 51-year-old male with a chronic history of hypertension, diabetes mellitus, and end-stage renal disease, secondary to diabetic nephropathy. He had undergone hemodialysis at our hospital. He underwent renal transplantation in a different country 18 months ago. The patient presented to our hospital with persistent hypertension, bilateral upper and lower limb swelling, and shortness of breath. He was on calcium channel blockers, beta-blockers, and diuretics. Vitally, he was hypertensive, tachypneic, tachycardic, and had bilateral limb pitting edema. Laboratory results showed high creatinine (295 mmol/L) and high uremia, and a chest X-ray showed flush pulmonary edema (Figure 1).

**Figure 2:**
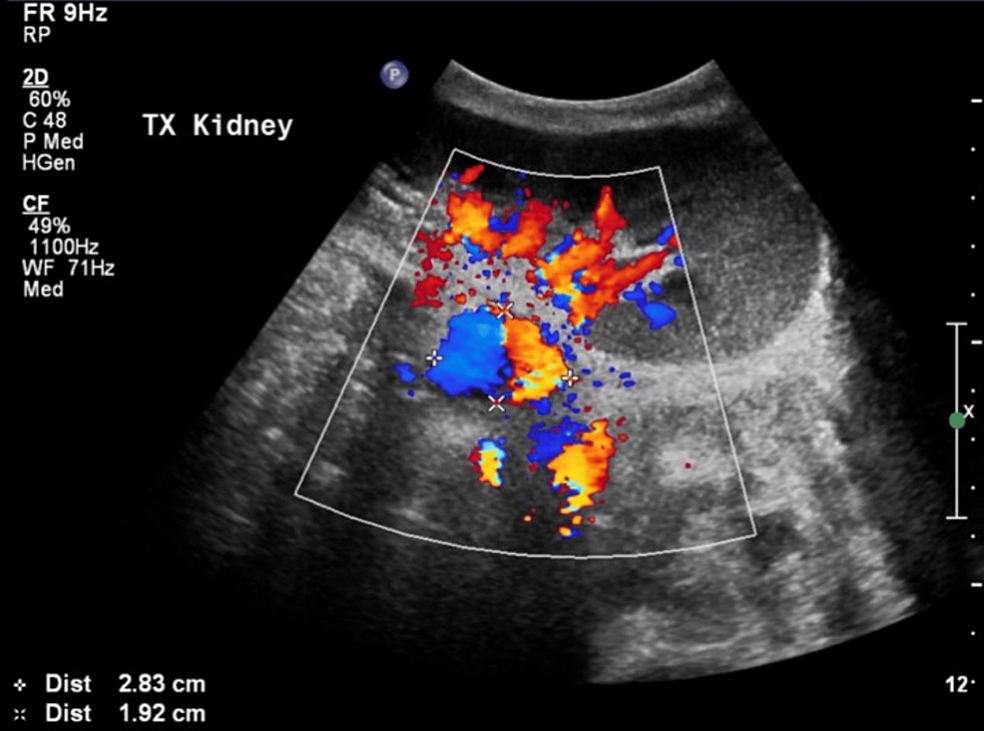
A pseudoaneurysmal sac measuring 28 × 19 mm.

Color Doppler sonography showed a pseudoaneurysmal sac 6 mm from the external iliac artery measuring 28 × 19 mm (Figure 2) compared to 17 × 18 mm seen in a previous study.

**Figure 3:**
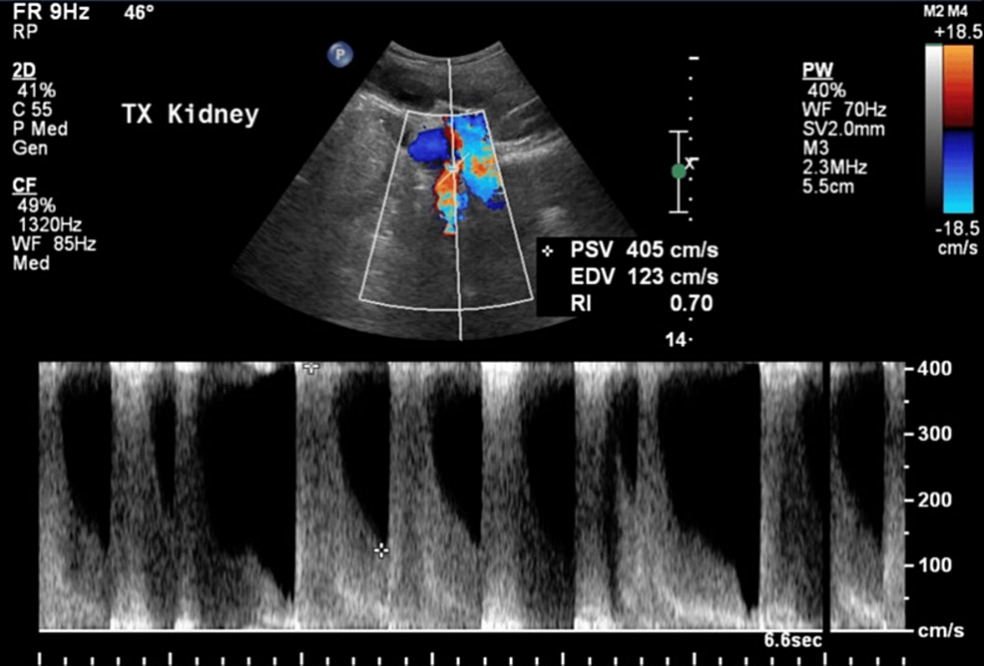
Stenosis at the anastomotic site showing a peak systolic velocity of 405 cm/s.

There was evidence of stenosis at the anastomotic site, with the external iliac artery showing a peak systolic velocity of 405 cm/s (Figure 3).

**Figure 4:**
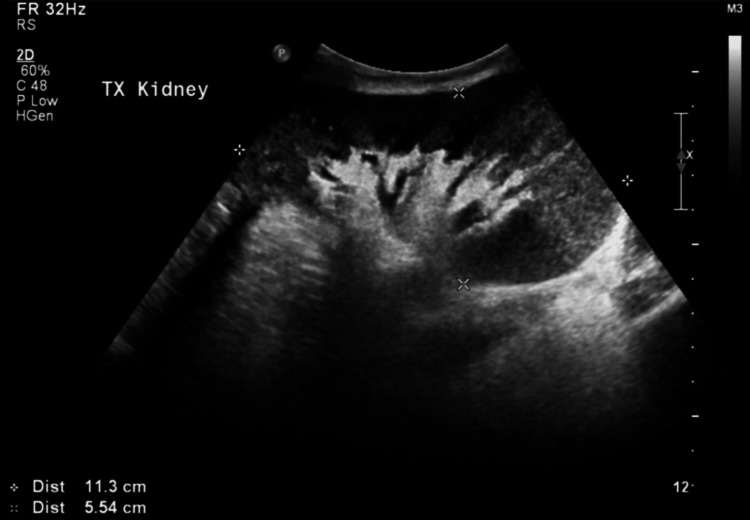
Transplanted kidney measuring 11.4 x 5.71 cm.

Ultrasonography was performed twice at an interval of 1.5 years, with both showing a normal allograft with normal blood flow. No evidence of hydronephrosis was found. The grafted kidney measured 11.4 x 5.71 cm and had remained unchanged (Figure 4).

**Figure 5:**
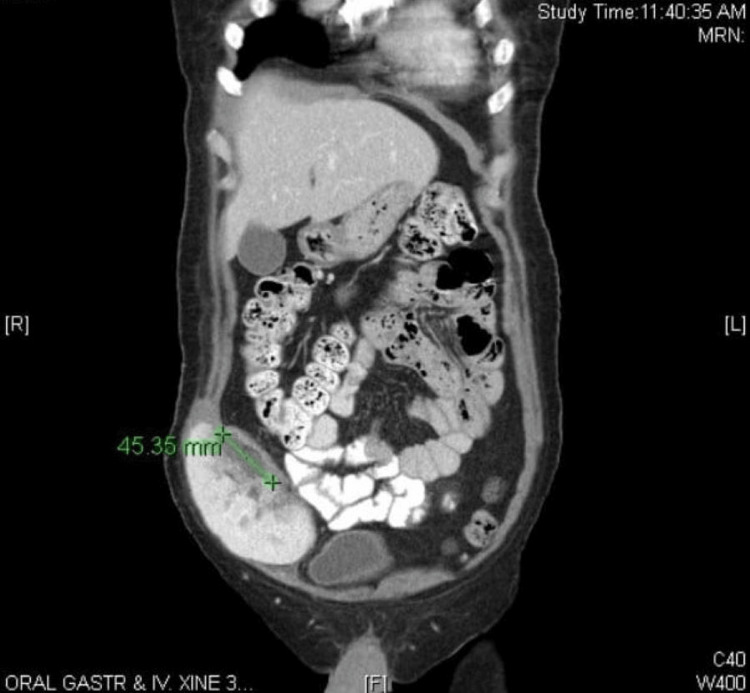
CT angiography of the pseudoaneurysm and anastomosis of the transplanted renal artery.

The origin of the pseudoaneurysm could not be determined using ultrasonography. Subsequent CT angiography confirmed a pseudoaneurysm and anastomosis of the transplanted renal artery with the right internal iliac artery, in addition to stenosis of the renal artery (Figure 5).

Given that the pseudoaneurysm of the renal artery graft occupied the entire length of the artery, with no distal landing zone to perform anastomosis after resection of the pseudoaneurysm by open surgical repair, the endovascular approach was the appropriate decision.

The postoperative course proceeded smoothly with a respiratory rate of 20 breaths/minute and oxygen saturation of 98% in room air. The patient’s renal function had improved. His creatinine level was 297 mmol/L at the time of admission and 99 mmol/L before discharge (normal: 71-115 mmol/L), urea was 14 mmol/L (normal: 2.5-6.4 mmol/L), sodium was 131 mEq/L, and potassium was 3.6 mmol/L. The patient’s chest also became clear, and the patient had orthopnea. The patient showed no further increase in hypertension.

## Discussion

Chronic renal disease is a growing problem in Saudi Arabia and worldwide. It is estimated to affect 9,892 per 100,000 people in Saudi Arabia. Renal transplantation is an ideal treatment strategy for patients with end-stage kidney disease [9,10]. Our patient’s transplant was a living-related donation. Due to regulatory policies in the Kingdom of Saudi Arabia, some patients travel abroad to undergo commercial kidney transplants. This leads to improper patient follow-up and, in general, more complications.

Vascular complications associated with renal transplantation include renal artery stenosis, arterial and venous thrombosis, arteriovenous fistula, intra-renal pseudoaneurysm, or extra-renal pseudoaneurysm. Our patient presented with renal artery stenosis, which may be a cause of graft dysfunction and hypertension, and extrarenal pseudoaneurysm, which is a rare but important vascular complication due to the risk of rupture [11]. The coexistence of both complications accelerates the rate of allograft loss.

Renal artery pseudoaneurysms following transplantation can be subdivided into intra-renal and extra-renal. They represent two different clinical entities, each with a unique etiology and prognosis. Most pseudoaneurysms are asymptomatic and are found incidentally on follow-up imaging [8]. Extra-renal types, which are our area of concern, are less common and can occur either at the surgical anastomosis as a complication of vascular reconstruction or infection or distant from the anastomosis as a result of mycotic aneurysm formation [12].

Different diagnostic tools, such as color flow Doppler, duplex Doppler scanning, CT angiography, MR angiography, and conventional angiography, can be used to identify false aneurysms and stenosis of the transplanted renal artery [13,14]. In this case, we used color Doppler sonography and ultrasound as the initial radiological imaging modalities. CT angiography was performed for further confirmation and evaluation of the pseudoaneurysm before embarking on the treatment modality.

The management of PAPRT is unclear with a case-by-case approach, with conservative management being the most practiced in asymptomatic, small, false aneurysms with no associated renal artery stenosis. The patient was educated that false aneurysms with a size of more than 2.5 cm increase the risk of rupture, which can be a fatal complication [15]. Renal artery stenosis, persistent hypertension, and elevated renal function are also indications for intervention. Open surgical repair and endovascular repair are currently reported treatment options for managing extra-renal false aneurysms with associated stenosis of the allograft in the iliac artery. Therefore, many minimally invasive procedures, such as percutaneous transluminal angioplasty and stent placement, are valid options for treatment and provide high technical success, as it is a safe and less invasive modality with a shorter length of hospital stay [16].

## Conclusions

Stenosis of the renal artery and superimposed pseudoaneurysm at the anastomosis with the internal or external iliac artery can be the leading cause of allograft loss, especially devastating allograft loss and the need for allograft nephrectomy, and should be kept in mind in kidney recipients with the presence of resistant hypertension, graft dysfunction, and high renal function. Percutaneous transluminal angioplasty and stenting of renal artery stenosis with or without pseudoaneurysms are the accepted treatment modalities in kidney recipients.
